# Wastewater Monitoring for Infectious Disease: Intentional Relationships between Academia, the Private Sector, and Local Health Departments for Public Health Preparedness

**DOI:** 10.3390/ijerph20176651

**Published:** 2023-08-25

**Authors:** Jeffrey L. Ram, William Shuster, Lance Gable, Carrie L. Turner, James Hartrick, Adrian A. Vasquez, Nicholas W. West, Azadeh Bahmani, Randy E. David

**Affiliations:** 1 Department of Physiology, School of Medicine, Wayne State University, Detroit, MI 48201, USA; avasque@med.wayne.edu (A.A.V.);; 2 Department of Biochemistry, Microbiology, and Immunology, Wayne State University, Detroit, MI 48201, USA; 3 College of Engineering, Wayne State University, Detroit, MI 48202, USA; wshuster@wayne.edu; 4 Law School, Wayne State University, Detroit, MI 48202, USA; 5 LimnoTech, Ann Arbor, MI 48108, USA; cturner@limno.com (C.L.T.);; 6 Detroit Health Department, Detroit, MI 48201, USA; 7 Department of Family Medicine and Public Health Sciences, School of Medicine, Wayne State University, Detroit, MI 48201, USA

**Keywords:** anonymity, collaboration, communication, dormitories, neighborhoods, nursing homes, pandemic, public health, SARS-CoV-2, COVID-19, sewersheds, wastewater

## Abstract

The public health emergency caused by the COVID-19 pandemic stimulated stakeholders from diverse disciplines and institutions to establish new collaborations to produce informed public health responses to the disease. Wastewater-based epidemiology for COVID-19 grew quickly during the pandemic and required the rapid implementation of such collaborations. The objective of this article is to describe the challenges and results of new relationships developed in Detroit, MI, USA among a medical school and an engineering college at an academic institution (Wayne State University), the local health department (Detroit Health Department), and an environmental services company (LimnoTech) to utilize markers of the COVID-19 virus, SARS-CoV-2, in wastewater for the goal of managing COVID-19 outbreaks. Our collaborative team resolved questions related to sewershed selection, communication of results, and public health responses and addressed technical challenges that included ground-truthing the sewer maps, overcoming supply chain issues, improving the speed and sensitivity of measurements, and training new personnel to deal with a new disease under pandemic conditions. Recognition of our complementary roles and clear communication among the partners enabled city-wide wastewater data to inform public health responses within a few months of the availability of funding in 2020, and to make improvements in sensitivity and understanding to be made as the pandemic progressed and evolved. As a result, the outbreaks of COVID-19 in Detroit in fall and winter 2021–2022 (corresponding to Delta and Omicron variant outbreaks) were tracked in 20 sewersheds. Data comparing community- and hospital-associated sewersheds indicate a one- to two-week advance warning in the community of subsequent peaks in viral markers in hospital sewersheds. The new institutional relationships impelled by the pandemic provide a good basis for continuing collaborations to utilize wastewater-based human and pathogen data for improving the public health in the future.

## 1. Introduction

The COVID-19 pandemic has manifested itself as ongoing, periodic, outbreaks of disease as numerous variants of SARS-CoV-2 evolve and spread. Each of these variants, including Beta (B.1.351 and descendent lineages), Delta (B.1.617.2 and AY lineages), and the rapidly mutating Omicron with its numerous subvariants (B.1.1.529, BA.1, BA.1.1, BA.2, BA.3, BA.4 and BA.5 lineages), have presented unique challenges related to virulence, infectiousness, symptomology, and vaccine evasion [1]. Urgency has driven research and development toward effective biomedical and social mitigation responses including vaccine development, use of antiviral medicines, masking, interpersonal distancing, increased ventilation, and detailed public health policies [2,3,4,5]. These responses have been integrated into a politicized sociocultural landscape that encompasses attitudes ranging from rejection of science-based evidence to pro-active adoption of mitigation, therapies, and implementation of public health guidance [6,7,8].

SARS-CoV-2, the virus that causes COVID-19, is shed in human feces [9] and appears in sewage before symptoms and clinical tests reveal outbreaks of the disease in a community [10,11,12,13]. In concert with testing initiatives, vaccination campaigns, and public health guidance, wastewater data have been used to warn of impending outbreaks [14,15,16]. When the number of SARS-CoV-2 RNA fragments decreases in a given wastewater volume, this decrease provides local agencies with vital context to adjust and reallocate resources toward mitigation strategies [17,18]. Furthermore, due to the common drainage of wastewater from residential to ZIP Code scales, this approach protects individuals from identification. Previously demonstrated benefits of screening wastewater for pathogen markers, such as polio [19,20], clearly supports the idea that screening wastewater for SARS-CoV-2 would likewise advance important public health goals.

Among the challenges in conducting wastewater-based epidemiology (WBE) is the need for close collaboration among multiple diverse disciplines and institutions. Accessing sewage, assaying its contents for pathogens, reporting the results to public health agencies and the public, and implementing public health responses involves multiple stakeholders that ordinarily are not in frequent communication. At the beginning of the COVID-19 pandemic, few institutions or scientists were regularly conducting monitoring activities of pathogens in wastewater. While many publications have since described data obtained from wastewater testing (for example, [21,22,23,24,25]), few have described the challenges encountered in developing the relationships needed for successful implementation. An exception is a 20-week study by Prado et al. [14] that briefly outlines their COVID-19 wastewater project, established at the initiative of a Brazilian municipality working in partnership with the authors’ local research institute and communicating results to the local Health Secretariat weekly. The sampling strategy, developed by the Municipal Health Secretariat, underwent changes as they recognized the need for more localized data than could be obtained sampling at treatment plants. The motivations for developing COVID-19-stimulated interorganizational collaborations have recently been the subject of a management study in Italy that observed that innovations were particularly contingent upon vertical and horizontal collaborations [26].

The objective of this article is to describe the “case history” about how new relationships among diverse institutions were developed and challenges were resolved for the purpose of conducting and applying COVID-19 WBE in Detroit, MI, USA. The institutions included a local health department (Detroit Health Department, DHD), an academic institution (Wayne State University, WSU), a private company (LimnoTech), and to a lesser extent, the local sewerage agencies (Detroit Water and Sewerage Department (DWSD) and the Great Lakes Water Authority (GLWA)), from whose facilities wastewater was collected. The WSU team came from diverse disciplines ranging from the College of Engineering (Civil and Environmental Engineering, as experts in wastewater infrastructure), the School of Medicine (molecular analysis of waterborne organisms), and the Law School (legal and ethical aspects). Our collaborative team faced numerous questions, including sewershed selection (large or small? Focused on neighborhoods, nursing homes, or health care facilities? Congregate housing or campus-wide?), communication of results (all data public? Some data restricted? Locations publicized or not?), and public health responses (encouragement or requirements for testing? Educational outreach?). Technical challenges included ground-truthing the sewer maps, overcoming supply chain issues, improving the speed and sensitivity of measurements, creating new informational dashboards, and training new personnel to deal with a new disease under pandemic conditions. This paper describes the development of this interdisciplinary and interinstitutional collaboration as a result of an intentional process in Detroit, impelled by the onset of a public health emergency and facilitated by an influx of government funding.

## 2. Development of the Monitoring Program

### 2.1. Site Selection and Collection

**Figure 1:**
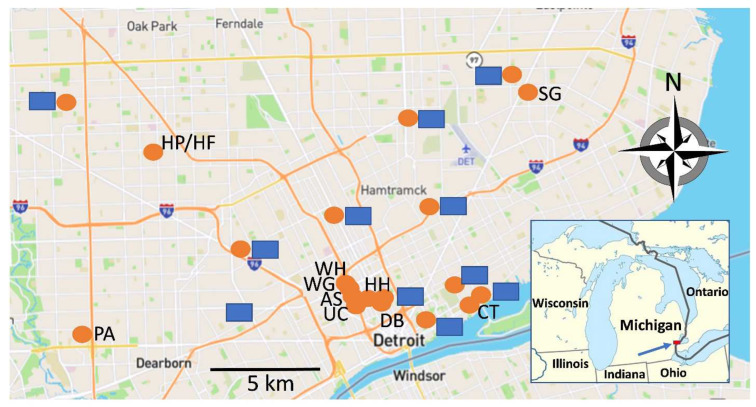
Wastewater sampling sites in Detroit, MI. The blue arrow and red marker in the inset show the location of the site map in Michigan. Collection sites are represented by orange circles, accompanied by either a black label or blue rectangle that obscures the site label for ethical considerations described in the text. Sites sampled for the EGLE-supported pilot project (2020–2021) are Wayne State dormitories, labeled WH, WG, AS, and UC, and adjacent dorm-sampling sites on campus not shown on the map; and hospital sewersheds HH and DB. The subsequent MDHHS-supported project (2021–2023) monitors the four labeled Wayne State sites and two hospital sites; several “ZIP Code” sites labeled PA, HP/HF (see text for explanation of the dual label), CT and SG; and small sewersheds that drain from long-term care facilities and surrounding neighborhoods.

In 2020, the Michigan Department of Environment, Great Lakes, and Energy (EGLE) funded a four-month pilot project to monitor dormitories on the WSU campus and nearby hospital sewersheds (Figure 1). This initial funding enabled the development of personnel training, laboratory upgrades and technical improvements, and strategic relationships with the DHD and LimnoTech in fall 2020 and winter 2020–2021, to safely collect and analyze sewage samples rapidly and accurately using digital droplet PCR (ddPCR) [27,28], LimnoTech had previously conducted wastewater research in small sewersheds with the School of Medicine team [29] and was therefore a natural partner for launching this project quickly with ample resources to collect samples over a wide geographic area. At the same time, DHD developed plans for how monitoring data could be translated to citywide public health actions. Concomitantly, the Michigan Department of Health and Human Services (MDHHS) partnered with EGLE to expand EGLE’s statewide network of academic and health department laboratories that had previously focused on beach water testing into an interactive group of 19 laboratories that refocused their surveillance targets on SARS-CoV-2 markers in wastewater.

The resulting monitoring programs and application of wastewater analysis technologies tracked on-campus concentrations of SARS-CoV-2 material in wastewater, and linked this data to outbreak potential. This process of wastewater-based epidemiology (WBE) proved in both principle and practice that the laboratories could provide quantitative data on the presence of SARS-CoV-2 markers in wastewater within 48 h of collection. Subsequently, beginning in June 2021, the MDHHS supported the statewide WBE network through expansion of the SARS-CoV-2 wastewater surveillance project in Detroit (Figure 1) and elsewhere in Michigan.

For the 2020 Pilot Project, sampling sites were selected on the WSU campus and several sewersheds draining local hospital centers. The larger scope of the 2021–2023 project built on the pilot sampling locations and represent several different types of congregate populations, scaling from single dormitories (hundreds of residents) to small sewersheds covering particular ZIP Codes (tens of thousands of residents). While many wastewater monitoring projects have focused on the influent to wastewater treatment plants (for example [18,24,30,31]), the WSU/DHD/LimnoTech team determined that public health responses may be better focused if the data were relevant to smaller sewersheds, where monitored populations can receive more direct and effective communication when heightened risks are detected.

Foundational to our effort was a reliable, fully equipped and staffed environmental services partner to assist in mapping, site scouting and selection, and conducting sample collections, a role that has been fulfilled by LimnoTech. LimnoTech collaborated in the development of the monitoring plan, acquiring and organizing multi-layered inputs of Detroit’s sewage catchments, population, and COVID-19 case densities to map medium-sized sewersheds citywide [32], that later formed the basis for the “ZIP Code” sampling sites in the 2021–2023 project. Subsequently, with field work and feedback from WSU, DHD, DWSD, and GLWA, LimnoTech mapped the smaller on-campus, hospital-center, and long-term-care facilities (LTCF-, also referred to as nursing homes) sewersheds, and the larger ZIP Code-associated wastewater collecting sites (Figure 1) for the expanded city-wide project.

Mapping the Detroit sewerage system was a challenge due to the many, sometimes contradictory, maps that have been compiled over more than a century of growth and aging of the sanitary and storm sewer systems of southeast Michigan. The City of Detroit is predominantly serviced by combined sewers in which the sanitary flow from housing and non-residential structures and storm flow runoff from impervious surfaces (e.g., roofs, streets) are combined in the same pipe. Consequently, the places where sanitary and storm flows become confluent is not always apparent, and the proportions of each type of influent are not defined quantitatively. Furthermore, many maps were created at various levels of resolution, and some are inconsistent with one another, possibly reflecting renovations that may have changed the connectivity among pipes and other parts of the network. Accordingly, we ground-truthed all sites and drainages, making note of wastewater conveyance physical data (e.g., pipe depths, flow direction, etc.).

The vagaries of sewer routing and drainage were another source of variability and adaptation in this project. For example, the initial map of sewersheds in ZIP Code 48235 indicated an accessible sampling site (HP) near the corner of Hubbell Ave. and Puritan Ave. at the southern (downstream) border of ZIP Code area 48235. As described later in this paper (Section 2.2.4, discussing ZIP Code sewersheds), despite maps that indicated that the manhole at site HP was sampling a sewershed with more than 25,000 people in it, a lower-than-expected level of SARS-CoV-2 at site HP during the Omicron surge (December 2021–February 2022) led us to map another site, HF, about two blocks north and 3 m deeper that we concluded was more likely to be sampling the target sewershed.

Discussions about potential legal and ethical concerns also informed our selection of sewersheds for wastewater sampling. While the results of tested wastewater samples cannot be linked to individuals, we remained mindful of privacy concerns that could arise from this sampling, particularly in smaller wastewater catchment areas [33]. Consequently, except in the case of the dormitory sewersheds, samples were collected from locations that could not be attributed to a single identifiable residence or facility. Even within the smaller sewersheds, results were publicly reported in a way that limited attribution of samples to specific facilities or neighborhoods. In addition, administrators of the LTCFs were notified that wastewater in their vicinity was being sampled.

The resultant field protocol involved weekly sampling conducted at the same time and on the same day, each week, except during weeks with holidays when sampling was usually delayed by one day. Wastewater collections were performed in the morning, in order to capture the morning diurnal peak of wastewater production between 8–10 AM. Wastewater was collected as a grab sample at each site, accompanied by a field blank collected approximately every tenth site randomly distributed on each collection date. Water quality indicators (pH, temperature, dissolved oxygen, conductivity, turbidity) were measured, along with flow measurements at a subset of the sites. If viral levels were high at a given site or set of sites, a follow-up sample within the same week was sometimes collected and analyzed. Methods used in the wastewater collections and analysis are described in detail in the Supplementary Materials.

### 2.2. Site Categories

We selected sewersheds that range in population size from a few hundred people to larger sewersheds that drain wastewater generated by between 3000 and 30,000 individuals, considered to be in the category of medium-sized regional sewersheds with small bore (i.e., small diameter, 0.3 to 1 m) sewers [34]. An additional consideration in selecting specific sampling sites was that the access point, generally a manhole, had to be safe from automobile traffic and other hazards. Our current set of sampling locations in order of increasing sewershed size includes the following:

(a) Small sewersheds with a focus on congregate facilities (LTCFs) in residential neighborhoods with at-risk populations;
(b) Sewersheds that receive wastewater from university housing (dormitories);
(c) Hospital center sewersheds;
(d) “ZIP Code-wide” sewersheds with multiple neighborhoods and businesses.

The rationale for collecting at each of these categories of sewersheds was as follows:

#### 2.2.1. Congregate Facilities with At-Risk Populations (LTCFs)

**Figure 2:**
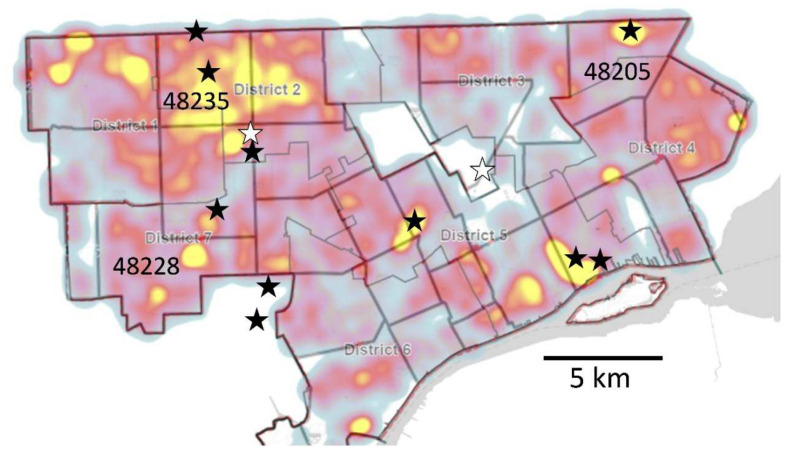
Locations of LTCFs and high disease rates in Detroit early in the COVID-19 pandemic. Black stars are the locations of LTCFs that had high levels of COVID-19 in May 2020; open stars had moderate levels. The purple/red/yellow background color is a heat map from a Detroit website of the period that showed COVID-19 prevalence in areas of Detroit. Yellow is the highest disease rate and often coincided with areas where nursing homes with high disease rates were located.

COVID-19 exhibited relatively greater mortality rates among the elderly [35]. Moreover, LTCFs were among the earliest settings in which SARS-CoV-2 infections in North America were detected [36,37]. Skilled nursing facilities (SNFs), a particular type of LTCF, have a high density of elderly residents that are susceptible to severe symptoms of COVID-19. Weaker immune responses and prevalence of comorbidities (diabetes, heart disease, respiratory disease, etc.) in the elderly, in addition to the congregate setting, made elevated mortality rates in SNFs an inevitability. Foreknowledge of a facility’s testing positivity rate is vital for health communications and mitigation efforts (e.g., vaccination and therapeutic drug treatments, testing, outreach and education, PPE supply, and investigations). In Detroit, several LTCFs had conspicuously high levels of infection early-on in the pandemic, as illustrated in Figure 2.

As a consequence of this observation, approximately half of our small sewersheds were chosen to include an LTCF as a significant contributor (between one-tenth to one-half of the sewershed population, based on census information). The decision was made early-on to sample a sewershed that collects wastewater flow from an area broader than sampling directly from the wastewater lateral that is specific to the LTCF. This was carried out to avoid potential stigmatization of a facility or community that shows high or increasing viral activity in the area (an ethical consideration; see [38]) and to simultaneously access a broader epidemiological perspective on the neighborhood in which they were located. LTCFs were notified that we had chosen a nearby public location to sample. Al-though all sampling occurred on public land, LTCFs were able to voice concerns with the DHD, if they chose. The DHD received no concerns, only positive reinforcement.

**Figure 3:**
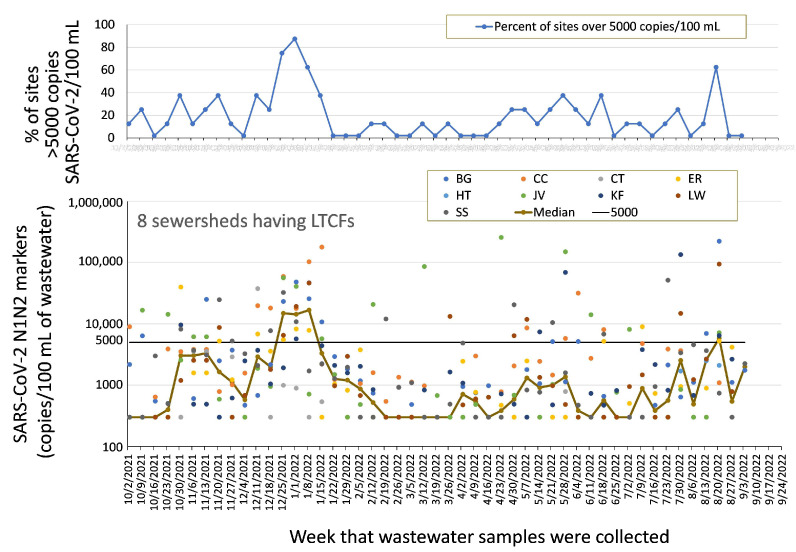
Levels of SARS-CoV-2 markers N1 and N2 (average of the two markers, measured in triplicate) measured weekly in 8 small sewersheds containing LTCFs, October 2021–September 2022 (nine labels are shown because the collecting site for one sewershed was changed (CT to HT) in August 2022). The upper graph shows proportions of the 8 LTCF sewersheds that had SARS-CoV-2 measurements above 5000 copies/100 mL. The lower graph shows values for each sewershed (points) and the median value of the 8 LTCF sewersheds (brown line).

Despite the best efforts of most LTCFs to prevent COVID-19 infections, Detroit facilities had frequent outbreaks of disease, as reported on a state case-enumeration website [39] and confirmed by our measurements of SARS-Cov-2 markers in LTCF-associated sewersheds (for methods, see Supplementary Materials). Figure 3 shows the level of SARS-CoV-2 N1 and N2 markers in wastewater across 8 LTCF sewersheds and the proportion of the 8 sewersheds that were considered to have high levels, which we quantify as exceeding 5000 copies/100 mL. Levels exceeding 100,000 copies/100 mL were observed on six occasions, as indicated by points in Figure 3 on 8 January, 15 January, 23 April, 28 May, 30 July, and 27 August. Using the median values on the graph as a guide, the data indicate that an outbreak occurred in November 2021. This was followed by a more extensive outbreak that peaked in early January 2022, subsided by late February 2022, and then was succeeded by sporadically rising levels beginning in May 2022. While we did not test for specific variants, the COVID-19 outbreaks observed in the monitoring area correspond to circulation of the Delta (B.1.617.2), initial outbreaks of Omicron variants (B.1.1.529, BA.1, BA.1.1, BA.2, BA.3) and since May 2022 more recent Omicron subvariants (BA.4 and BA.5) occurring globally, as reviewed in [40] and observed in [41,42].

#### 2.2.2. Congregate Facilities: University Housing (Dormitories)

**Figure 4:**
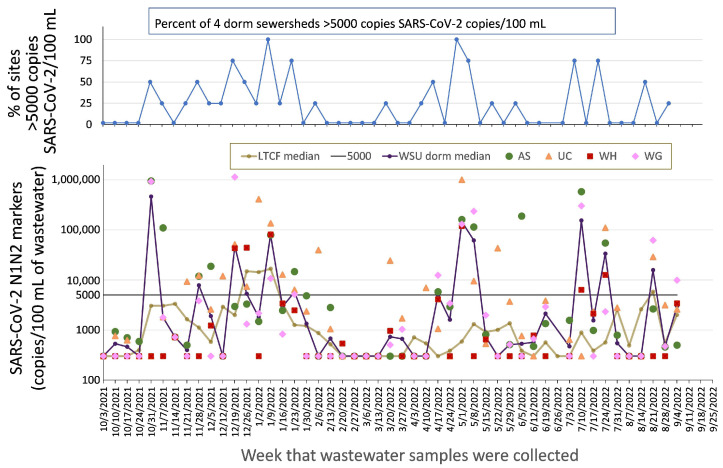
Levels of SARS-CoV-2 markers N1 and N2 (average of the two markers, measured in triplicate) in wastewater that was collected weekly from sites AS, UC, WG, and WH that drain from WSU dormitories (locations shown in Figure 1). The upper graph shows proportions of the four sewersheds that were above 5000 copies/100 mL. The lower graph shows values for each sewershed (points), the median value of the 4 WSU dormitory sewersheds (purple line), and, for comparison, the median line for the 8 LTCFs illustrated in Figure 3 (brown line).

Complementary to the LTCF strategy was a focus on university congregate living facilities, i.e., student dormitories. Several early reports provided evidence that early detection of an outbreak via wastewater could effectively be used to trigger mass testing within a dormitory, isolation of infected individuals, and, circumstantially, prevention of a larger outbreak [43,44]. With support from the administration of WSU, our pilot project focused on detecting viral markers in wastewater from several on-campus dormitories. In the subsequent citywide project, four dormitory sewersheds were selected, based on the sensitivity with which they had detected viral markers in the pilot project (sites shown in Figure 1). The original university housing sampling plan, developed in collaboration with the Campus Health Committee, was to use wastewater data to inform the required testing of high-risk subpopulations within the dormitories. Wastewater data were further integrated with modeled data for the Detroit metropolitan region, to determine best mitigation practices (masking, social distancing, virtual-, in-person, or hybrid in-person/virtual instruction) that would be recommended or required of WSU students, staff, and faculty. The SARS-CoV-2 data from the four WSU dormitory sites selected for the 2021–2023 project are illustrated in Figure 4.

A complication to the interpretation of this dormitory data is that guests or visitors who do not otherwise live in congregate housing contribute to the wastewater stream. Although the predominant sampled population was likely dormitory residents, wastewater may also come from guests or several businesses, as well as the Campus Health Center, located in the same buildings.

While a focus on specific resident facilities at a university (WSU) has the same ethical concerns of privacy and stigmatization as aforementioned regarding LTCFs, university administrators reasoned that these populations of mostly young, healthy people had chosen to attend college and live in college dormitories as a voluntary activity governed by agreed-upon rules. Consultation with the WSU Institutional Review Board (IRB) confirmed this judgement. Therefore, the imposition of monitoring and mitigation rules during a public health crisis by the institution itself (i.e., not imposed on it by an outside agency) would be within acceptable ethical bounds.

A further consideration was that due to the large commuter population among WSU students and the potential for infections among staff not residing in dormitories, dormitory wastewater data may omit a large campus subpopulation. Accordingly, we have recently begun to sample sewers that receive wastewater from classroom, office, and laboratory buildings. 

For comparison, Figure 4 also includes the median curve for the 8 LTCFs (same median curve as in Figure 3). SARS-CoV-2 levels in wastewater from the WSU dormitory sites often exhibited higher concentrations of the SARS-CoV-2 marker signal than the wastewater from sewersheds that included LTCFs and also a somewhat different pattern of SARS-CoV-2 presence than LTCF sewersheds. Seven samples from the WSU dormitory sewersheds exhibited levels of SARS-CoV-2 markers greater than 300,000 copies/100 mL, a level greater than any sample from the LTCF sewersheds (significantly different, Fisher exact test, *p* < 0.001). High level detections in the WSU dormitories may have been affected by the academic calendar and by “social leave” times around Thanksgiving (24–28 November 2021), before and after Christmas/New Years break (22 December 2021 to 9 January 2022), spring break (12–20 March 2022), and start of the spring (6 May 2022) and summer sessions (29 June 2022).

#### 2.2.3. Hospital Center Sewersheds

Among the largest challenges of the COVID-19 pandemic, especially prior to the advent of COVID-19 vaccines, was under-capacity in healthcare centers when outbreaks reached their peaks. Early mitigation efforts and messaging were designed to “flatten the curve” or proactively manage the spread of the virus and infection with COVID-19. This effort was also hoped to prevent shortages of PPE, equipment, beds, and staff, as well as to prevent healthcare worker burnout. Sampling hospital sewersheds would give an additional, corroborative measure of COVID-19 impact on the local healthcare system. We sampled hospital sewersheds in the early days of the pandemic as a positive reference site wherein we were assured a positive signal. This approach was practiced as we optimized the sensitivity of our analytical methods and determined that our tools were sensitive enough to see a signal in small sewersheds. Accordingly, we sampled two Detroit hospital sewersheds near Wayne State University from the earliest days of this project.

**Figure 5:**
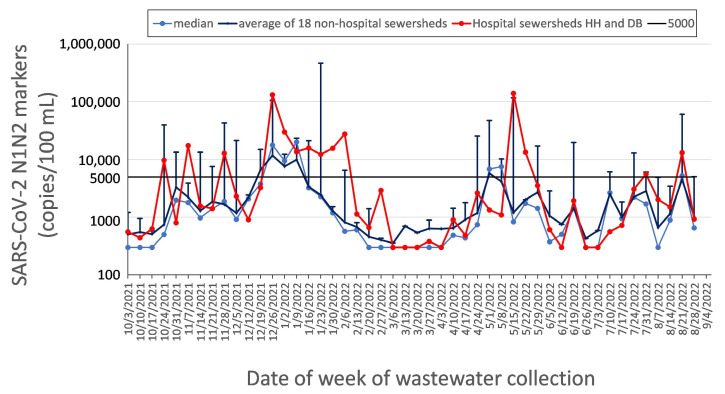
Comparison between hospital center SARS-CoV-2 wastewater levels (average of sites HH and DB, red line) and the average (mean ± SE, dark blue lines with error bars) and median (light blue line) of 18 other sewersheds sampled across Detroit.

Figure 5 shows that the onset of new citywide outbreaks (considered to be large increases from low- or mid-range baselines of the 18 non-hospital sites that were sampled) often preceded peaks in hospital sewersheds by several weeks. Indeed, the levels detected in the hospital wastewater sites correlated best with the median levels measured citywide in the non-hospital sites one and two weeks earlier, with R^2^ = 0.39 and 0.35, respectively; *p* < 0.001 (illustrated graphically in Supplementary Materials, Figures S1 and S2). Together with clinical testing data and epidemiological models, these predictive data have the potential to be used for logistical and capacity planning not only for the sampled hospital sewersheds, but also for other hospital centers in Detroit.

#### 2.2.4. “ZIP Code” Sewersheds

We adapted and evolved our sampling strategy in response to a recommendation from DHD to focus on sewersheds that would sample larger populations. The population estimates of ZIP Codes 48228, 48235, and 48205 are 57,700, 46,400, and 33,600, respectively. Each of these ZIP Code areas had relatively high case counts of COVID-19 early in the pandemic. For ZIP Code level sampling, we identified manholes that met two criteria: (1) the access point would provide wastewater from populations of at least 4000 residents; and (2) drainage areas would be either completely contained within the ZIP Code area, or extend less than 10% outside of its designated sewerage area. Monitoring ZIP Code-level sewersheds provide a sampling area that fills a gap between the neighborhood and the city-wide flow in large interceptors that deliver wastewater to the wastewater treatment plant. At the largest scale of monitoring, influent at the Detroit Water Resources Recovery Facility (WRRF) comes from >3 million residents and commercial-industrial sources across the southeast Michigan region, and arrives at the plant in three interceptor flows. Sewage transit times, from original source to arrival at the WRRF are as long as 24 h for the wastewater flows traveling from the outskirts of the service area and might enable detection of an outbreak anywhere in its service area without identifying where. By contrast, the ZIP Code-sized sewersheds that we sampled enable a more focused indication of the areas in the city in which outbreaks are actively occurring.

To explore this concept further, we hypothesized that with the larger population and range of flow travel distances for ZIP Code-sized sewersheds, the copies per unit volume measured would vary less than for the much smaller LTCF and dormitory sampling sites. We confirmed this hypothesis by comparing the probability of a measurement above the limit of detection (LOD) in the ZIP-Code sewersheds (mean ± SD = 91% ± 4% for the 3 ZIP Code sites) to the 8 LTCF smaller sewersheds (66% ± 14%; significantly different at *p* < 0.001, unpaired *t*-test with Welch’s correction for unequal variances). Despite fewer detections, when measurable levels were detected, week-to-week percent change in monitoring data in LTCF sewersheds were larger than any of the ZIP Code sites. For measurements exceeding the LOD, the median week-to-week percentage change was 240% for the ZIP Code sewersheds and was considerably less than the 350% observed for the LTCF sewersheds (*p* < 0.0001, Mann-Whitney test).

**Figure 6:**
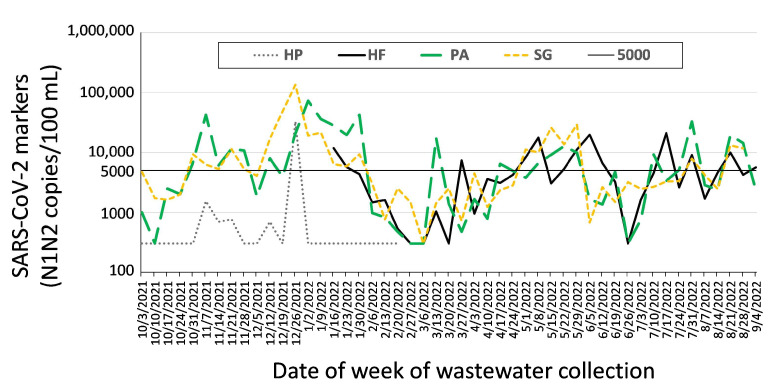
Data supporting the change from site HP to HF for monitoring ZIP Code 48235. Locations of the sampling manholes, HF and HP, are shown in Supplement Figure S3. Data from HF, HP, and two other ZIP Code-associated sewersheds, PA and SG are shown. HF correlated better than HP with the other ZIP Code-associated sewersheds. The dashed blue box shows the boundaries of ZIP Code 48235; black lines indicate the sewers.

A problem that we encountered for ZIP Code 48235 was that wastewater from our initially mapped site, HP, had less SARS-CoV-2 signal than expected during the Omicron surge (December 2022–February 2023). Prior to the Omicron surge, site HP was typically reporting much lower SARS-CoV-2 marker levels than the other ZIP Code sites in the city (Figure 6). During the Omicron variant surge, very little N1 and N2 SARS-CoV-2 markers were detected at HP, except on the peak day of 29 December 2021, as illustrated in Figure 6. In mid-January 2022, we decided to add an additional collection site, HF, about two blocks north of site HP (Supplementary Materials, Figure S3). The sewer pipe at HF was approximately 3 m deeper than at site HP. The new site was sampled for 5 weeks on the same day, and similar time of day to site HP, and consistently showed measurable SARS-CoV-2 marker levels, comparable to those that were reported at the other ZIP Code sites. With consent from DHD and MDHHS, we changed our collecting site for the 48235 ZIP Code sewershed to site HF.

The fact that site HF is “upstream” (i.e., north) of site HP, but 3 m deeper, suggests that site HP is likely a sub-sewershed within the same basin, and not sampling the larger area that we had mapped (water doesn’t travel “uphill”). Furthermore, since our goal was to sample from sites most likely to give early warning of a potential outbreak, collection from site HF was preferred since we consistently detected higher virus levels at HF than HP, when the disease was reported to be in the community according to clinical data.

ZIP Code-level monitoring provides data at a neighborhood-community level, where greater attention can be paid to vulnerable populations while maintaining anonymity, which is a key attribute of wastewater-based epidemiology [38]. Our focus on these intermediate-sized sewersheds enables a more rapid adaptation to changing pandemic conditions and the development and implementation of health responses along political (e.g., city council districts) or EMS/healthcare system-based subdivisions, wherein each actively collaborates with the Detroit Health Department.

#### 2.2.5. Potential Future Collection Sites

Since the inception of our project, we have been mapping sewersheds that drain geographical areas that were not included in the original sampling plan, as they were not identified as neighborhoods with high COVID-19 rates early in the pandemic. Several “unsampled” areas are evident in the map in Figure 1, in the north-central and southwest areas of Detroit. The north-central area has been a particular challenge as the sewersheds are either relatively small laterals with low populations (<3000 individuals) or along the “main line” draining from >100,000 people in Oakland County, north of Detroit, an area under the surveillance aegis of Oakland County.

## 3. Adoption and Improvement of Laboratory Analysis

The goal of the laboratory analytical techniques was to purify and detect SARS-CoV-2 RNA, and to communicate the results rapidly to the Campus Health Committee and DHD. Necessary steps included the concentration and purification of RNA in wastewater, purifying it sufficiently that PCR inhibitors in wastewater would not prevent PCR techniques from detecting the virus, if present. Previous evidence suggested that clinical test PCR targets developed by the Centers for Disease Control and Prevention (CDC) [45] could also be detected in wastewater [46]. Our team adapted the CDC assay to detect SARS-CoV-2 RNA by purifying wastewater RNA by a standard RNA extraction and purification technique (PEG/NaCl followed by Trizol purification, as described by Wu et al. [47]), and detected the N1 and N2 SARS-COV-2 sequences in WSU dormitory wastewater with the CDC probes using conventional qRT-PCR in November 2020.

To promote the adoption of similar techniques for monitoring SARS-CoV-2 in wastewater across Michigan, EGLE purchased Bio-Rad ddPCR systems for 19 participating laboratories across the state. At the limit of detection (LOD), ddPCR was estimated to possess approximately 10 times the sensitivity for detecting SARS-CoV-2 as qRT-PCR [48]. Furthermore, ddPCR was more resistant to the effects of PCR inhibitors and enabled quantitation of SARS-CoV-2 gene copy number without the need for a calibration curve. Our team also adopted the PEG/NaCl concentration method (requiring the purchase of large volume refrigerated centrifuges for several laboratories, including at WSU), but with an alternative Qiagen minicolumn procedure [49] for purifying RNA.

When citywide sampling began in June 2021, WSU sought to improve concentration and purification steps to begin ddPCR and report results sooner. As described in West et al. [28], we compared automated magnetic-bead-based systems by three manufacturers to each other and to the PEG/NaCl/Qiagen method. As a result, the WSU team selected the Chemagic 360 instrument with a 12-sample head, based on the following criteria: (A) the Chemagic method required only 10 mL of wastewater compared to 100 mL for the PEG/NaCl/Qiagen method. (B) the time to purify RNA from wastewater is less than 2.5 hr, approximately half the time needed for the PEG/NaCl/Qiagen method. (C) Although the cost for supplies per sample is only about $20 USD for the PEG/NaCl/Qiagen method, $30 USD for the Chemagic 360, and $40 USD for the other two automated methods, taking into account the lower labor needed for processing with the Chemagic 360, the per sample cost for processing was less with the Chemagic 360 than the other methods.

Perhaps the most critical consideration was that using the Chemagic 360 improved the sensitivity with which SARS-CoV-2 in wastewater could be detected, as exemplified by improved RNA recovery of both an internal “spiked” standard, Phi6, and the quantity of N1 and N2 markers detected per sample. A direct comparison of Phi6 from the same samples showed (D) an approximately 4-fold higher recovery by the Chemagic 360 system than the PEG/NaCl/Qiagen method. Similarly, (E) the number of copies of SARS-CoV-2 detected per 100 mL in the Chemagic-purified RNA samples was consistently higher, averaging 4.9 ± 3.4 times the number of copies measured in the same samples purified by the PEG/NaCl/Qiagen-method, and for some samples, the Chemagic method averaged more than 10× the signal detected in the same samples purified by PEG/NaCl/Qiagen [28].

**Figure 7:**
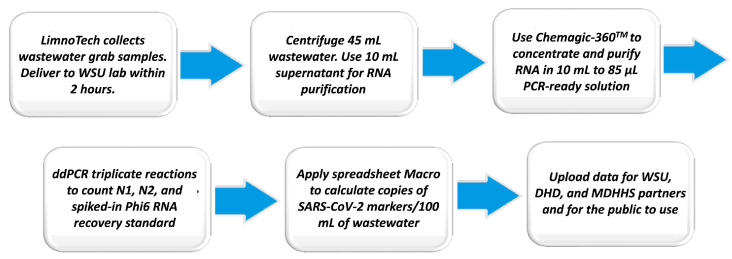
Summary of LimnoTech and WSU wastewater processes to collect wastewater, purify RNA, analyze for SARS-CoV-2 viral markers and distribute data. The entire process usually takes less than 36 h from wastewater collection to data distribution. Detailed methods are provided in the Supplementary Materials.

As a result of the above considerations, the method used since October 2021 (hence, for all of the data reported in Figure 3, Figure 4, Figure 5 and Figure 6) was the Chemagic method, described in the Supplementary Materials to this paper. The current sample collection and analytical methods flow is summarized in Figure 7.

## 4. Communication and Public Health Actions

### 4.1. Data Presentation and Reporting

The WSU team built a secure website (https://www.ramlabwsu.org/sars-cov-2-monitoring.html, accessed on 28 June 2023) for prompt reporting of data to the public, and through password-protected “back pages”, to the DHD. The site also maintains a readily accessible archive of past data. Additionally, as part of the statewide laboratory network, all data, including additional quality control data (e.g., recoveries of spiked-in internal standards, which are not part of the public and local dashboard uploads) were uploaded via secure FTP and Microsoft Teams connections to state-maintained databases, from which some data were also forwarded to the CDC.

Data from WSU dormitories, hospital center sewersheds, and ZIP Code sewersheds were considered unrestricted; however, data from small sewersheds with identifiable LTCFs were designated confidential and restricted (i.e., not reported publicly). This decision was a negotiated outcome with DHD. This critical engagement between the academic and LHD partners speculated on limiting the public identification of nursing homes that possibly had high COVID-19 caseload. This collaborative engagement led to changes in sample reporting such that LTCF site ID nomenclature had anonymous, non-traceable designations, that maps of sampling sites should distort the proximity of sampling sites to the LTCF source, and that data from individual LTCFs should be restricted. Once these reporting details were settled, DHD agreed on our reporting data to the state-maintained databases.

For data analysis and state reporting, the project used a task-specific Excel MACRO program (programmed by collaborators in the statewide project) to extract and average triplicate data from samples and controls from the .csv file outputs of the Bio-Rad ddPCR. Two distinct output spreadsheets from this MACRO were uploaded via FTP and Microsoft Teams for the state network dashboard and analysis team.

The public side of the WSU web-based dashboard provides both interpretative context and quantitative data for wastewater SARS-CoV-2 marker data. At the time of publication (July 2023), this dashboard includes a log-scaled graph that integrates data from all sites in the City with no identification of the locations or boundaries of the sewersheds being sampled, a list of the weekly percentage of positive and “high positive” (above 5000 copies/100 mL) sites, a short paragraph that is updated weekly interpreting recent data as rising or falling and indicative of widespread infections (many sites/high levels) or low level localized (without indicating the locality) infections. This method of presenting data for the public provides order-of-magnitude alerts for increases in SARS-CoV-2 marker levels that might indicate impending or ongoing outbreaks. For information about locations and data from specific sites, the public is referred to the state’s public dashboard (https://www.michigan.gov/coronavirus/stats/wastewater-surveillance/wastewater-surveillance-for-covid-19, accessed on 28 June 2023), which displays data for all unrestricted sites, individually.

The password-protected pages of the WSU reporting dashboard were designed for prompt, confidential reporting of results (i.e., within 48 h of sample collection). The Excel MACRO program Teams spreadsheet was pasted into a WSU data display spreadsheet that identified samples at the limit of detection and organized the data into a compact, readable table, and graphic display. The table and graphs were then uploaded into the website engine. A short summary of the week’s data, highlighting “sites of concern” was inserted, and an e-mail notification about the upload was sent to the Epidemiology and Population Health Sciences division at the DHD and the WSU Campus Health Committee.

### 4.2. Epidemiology and Data Response

At WSU, notifications sent to the WSU University Campus Health Committee sometimes triggered the Committee and staff to develop university health responses. At the DHD, wastewater data were received and confirmed by the Chief of Epidemiology and Population Health Sciences. Each entity discerned for itself its own data action thresholds, considered in context with other available information, including clinical testing data, vaccination rates, hospital capacity, recommendations from the CDC, etc. These collaborators made data-based decisions that result in public health actions, that have included:

- Focused, increased testing within sampled populations;
- Targeted vaccination campaigns or enhancement of capacity for existing programs;
- Encouragement of protective actions and cultural practices (social distancing, masking, hand-washing, etc.);
- Communication of potential higher risk to affected populations;
- Related resource planning in anticipation of a potential new outbreak;
- Investigations of clinical infection data at or near sites from which high marker levels have been reported.

**Figure 8:**
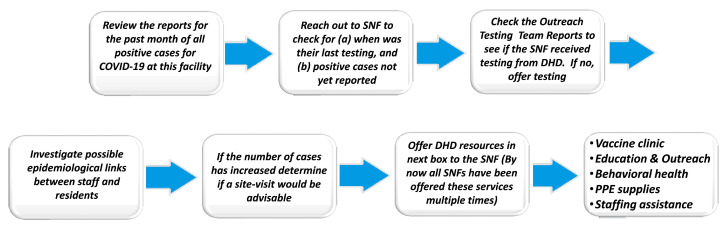
Summary of information and resources process flow by DHD in response to wastewater testing data provided by their academic partner and other epidemiological data.

The schematic in Figure 8 summarizes the information flow at DHD that has resulted in improved testing and vaccination responses at a number of SNFs. When WBE data indicated elevated levels of viral markers, DHD personnel made timely calls to the high-risk facilities in the affected sewersheds, alerting them, making suggestions, and encouraging a greater frequency of reporting of required case data. The outreach included formulating a letter that was sent out to nursing home managers-operators when high levels of SARS-CoV-2 viral material were detected in their servicing sewershed. DHD made prompt calls to the facilities when wastewater data indicated elevated levels of SARS-CoV-2 in their neighborhood. As a part of making positive contact with the facility manager, DHD staff made suggestions on how to deal with management of COVID-19, and also requested increased frequency of reporting of case data. In order to better engage and create forums for factual communication, DHD created a presentation at the DHD 2020 annual Block Party to introduce residents to how the communications process worked. At the same time, both DHD and WSU worked with local news media through which regular news spots ensured that a broader audience would know of these local efforts in an easy-to-assimilate context.

A specific example of a potential impact of the alerts generated by our wastewater data is an observation at site JV, an LTCF-associated sewershed. Site JV exhibited sporadic, but occasionally very high (e.g., over 250,000 copies/100 mL on 24 April 2022; see green dots in Figure 3) marker levels during March and April 2022, when most other LTCF sewersheds exhibited only low levels of markers. The WSU team highlighted this unusual behavior in its reports at the end of April along with one additional fact: based on state reporting of resident and staff cases at the LTCF in the JV sewershed, no COVID-19 case had been self-reported by the facility since 18 August 2021. The DHD’s Epidemiology and Population Health Sciences Division provided this information to its Testing and Investigations unit. Subsequently, the LTCF in the JV sewershed began to report both resident and staff cases to the state database (e.g., cumulatively between 25 May and 31 August 2022, more than 30 resident and 10 staff cases were reported). The increased reporting may have resulted from the involvement of Testing and Investigations and could potentially have improved the LTCF’s mitigation, testing, and vaccination efforts. Site JV SARS-CoV-2 marker levels have been below the median for the 8 LTCFs for 70% of measurements since 1 June 2022. High levels at particular LTCF-associated sewersheds are always highlighted on the secure data dashboard and follow-up communications and have triggered outreach to the associated LTCF to determine if the signal may indicate a need for enhanced vigilance or resources.

Similarly, at WSU, a rapid rise in wastewater levels of SARS-CoV-2 on 31 October 2021 at sites AS and WG (see Figure 4) triggered Campus Health to request that students in the dormitories to receive PCR testing. Testing was voluntary and incentivized by $20 gift cards (“Get tested, get 20”) and mostly occurred more than 5 days after the original finding of elevated SARS-CoV-2 marker levels. 148 students (approximately 20% of the dormitory population) were tested and a positivity rate of 0.7% was reported, which was considered low. At the same time, the regular testing program on campus (which conducted tests of any student or employee with symptoms who voluntarily requested a test or was required to test weekly due to obtaining a vaccination waiver) was exhibiting an increasing level of infections on campus (specifically, a rise of confirmed and presumptive cases from 47, the week before 31 October 2021, to 64 the week of 31 October 2021, to 70 during the second week of November 2021). It is unknown whether the increased publicity about COVID-19 and testing increased student mitigation efforts and potentially decreased the magnitude of this outbreak.

Comparisons of dormitory SARS-CoV-2 wastewater levels to campus case prevalence during the subsequent Omicron outbreak (approximately December 2021 to February 2022) revealed a correlation of marker levels with confirmed and presumptive cases two weeks later, with an R^2^ = 0.7. On the basis of predictive models and general disease levels in the community at the time, the University restricted campus access and changed classes to virtual for part of that period but did not mount an incentivized campus-wide testing program as they had in November 2021.

### 4.3. Public Communication, Education, and Outreach

In addition to reporting on the MDHHS and WSU data dashboards, the WSU team has conducted communication activities specifically related to public information about wastewater epidemiology. Led by laboratory communication assistants, these activities have included talks to student groups (Med-Direct students, WSU Faculty Senate, etc.), presentations to community groups (WSU Community Engaged Research Symposium, Flint COVID-19 Community Webinar), and tabling at local gatherings (e.g., at a weekly farmers market during October 2022).

In order to better engage and create forums for factual communication, DHD created a presentation at the DHD 2020 annual Block Party to introduce residents to how the wastewater communications and other epidemiological processes worked. At the same time, both DHD and WSU worked with local news media resulting in the project being featured in television news reports [50]. These efforts have supported the goal of ensuring that a broader audience would know of these local efforts in an easy-to-assimilate context.

## 5. Discussion

As a case study, this paper adds to the body of literature on use cases of wastewater surveillance that have been encouraged for guiding and increasing confidence in future WBE applications [51]. This paper reviews the development of collaborative relationships and communication pathways among three different sector organizations in Detroit, MI—an urban-serving university, a local health department, and an environmental consulting company—that came together to collect, analyze, disseminate, and act upon wastewater-based SARS-CoV-2 epidemiological data. Our experience agrees with the conclusions of a study of 25 colleges and universities that began WBE for SARS-CoV-2 at the beginning of the epidemic that collaboration—both within and outside of the institution—was considered essential in nearly every case [27]. Collaboration required trust-building among the collaborators, alignment of goals, mutual understanding of any concerns, development of reliable communication, and ongoing agility as technology, policy, and resources shifted over the first two years of the pandemic. Trust-building and alignment of goals have been identified as important features in establishing collaborations that produced innovative outcomes in response to the pandemic [26,52]. The collaboration with LimnoTech followed a pattern highlighted by Greco et al. [26] in which vertical interorganizational collaboration (i.e., collaboration with existing suppliers or customers) was a facilitator of COVID-19 related innovations.

Collaboration and communication with public health authorities, the users of the wastewater data, was also necessary for success. A review of WBE efforts in Canada noted especially the necessity of the buy-in of public health authorities early in the process of building an impactful wastewater testing program [53]. A campus monitoring study in Maryland also concluded that stakeholders should collaborate early in the process to decide on target populations and what potential actions may be taken in response to positive results. In Michigan, collaboration with the Michigan Department of Health and Human Services was facilitated by a pre-existing network in another department of the State of Michigan (Environment, Great Lakes, and Energy, EGLE) that repurposed itself from beach water testing to focus on wastewater testing. EGLE also invited additional institutions (including ours) to join the network. Similarly, the early involvement of DHD in developing the sampling program in Detroit was critical to success in focusing our WBE efforts on small and medium-sized sewersheds.

Challenges were addressed as rapidly as possible through outreach and communication. The result of intensive discussions particularly at the outset of these collaborations enabled the timely adoption of novel sampling and analytic techniques and the resolution of how to best protect privacy, promote equity, and safeguard the autonomy of individuals, organizations, and neighborhoods. Such efforts informed the appropriateness of public health response modalities, with the goal of reducing the severity of outbreaks, guided by the weekly rise and fall of wastewater signals and other pandemic-related data.

Since some of our samples represented neighborhoods in particular areas of the city, we considered how the analysis and release of data we collected could impact community members [33]. Diverse opinions exist about the minimal sewershed size that may be ethically assayed or reported. One study suggested a minimum sewershed population should be >10,000 people [54]. The CDC wastewater reporting network says it will only post data on its COVID Data Tracker for sewersheds that are >3000 people [55]. In contrast, at least one study reported using SARS-CoV-2 wastewater data from sewer pipes attributed to as little as one floor in a 5-story dormitory and as few as 45 people [56]. Exposure of COVID-19 infection status can have negative implications for privacy of both individuals and groups, who may be subjected to discrimination and stigmatization based on the disclosure of this information [57,58,59]. Privacy risks can be minimized and understood as a tradeoff, by way of the demonstrated public health benefits of SARS-CoV-2 WBE surveillance efforts [60]. In places where individual testing may be unavailable, or problematic due to structural or social barriers to health care, wastewater detection of SARS-CoV-2 may justify greater attention from public health authorities and allocation of resources to affected communities that would otherwise be overlooked [61].

Through our collaborative wastewater screening and epidemiology efforts in Detroit, we learned that in designing protocols to apply to infectious diseases, it is important to consider how wastewater-based epidemiology is perceived by members of the communities being served, and to inform and consult with community members. Without community buy-in and a belief in the credibility of the science, most mitigation efforts are of little use. The conditions for community outreach were complicated by pandemic-related fears and untested routes of communication. From the academic partner side, we worked within the MDHHS network to provide a central, public-facing website to disseminate data and information cleared at the state level. Our regular, weekly presence in at least 20 neighborhoods provided some degree of visibility and interaction during the pandemic. However, due in part to initially high turnover in personnel and difficulty in locating replacements, our Community Outreach and internet reporting sites were not as well-developed as we had hoped. Nevertheless, as described above in Section 4.3, we hosted presentations in various WSU-area venues, and stories in national and local media about WBE gave further support to our efforts to communicate these data to the public. Through online weekly statewide wastewater testing network meetings, we shared insights and challenges with other laboratories and collaborated with colleagues involved in nationwide WBE efforts.

The data-reporting dashboard for communicating with our partners has worked well, and the public-facing parts of our dashboard are relatively simple; however, according to web site statistics these sites are viewed fewer than 100 times per month. We have provided graphic data quantitatively on a logarithmic scale in order not to obscure significant increases from baseline that otherwise would appear insignificant on graphs that also include highest detected levels that are orders of magnitude higher. In comparison, a review of more than 100 SARS-CoV-2 wastewater epidemiology dashboards found that the majority of such sites display data on a linear scale and only a quarter use a logarithmic scale [62]. Ref. [62] suggests that data should be displayed on both linear and logarithmic scales, with possibly a toggle between. We have favored a simple, brief page; whereas other websites, including the site to which our unrestricted data are uploaded for the state of Michigan, require clicking through multiple levels to access maps, determine site locations, view data, and understand trends. Elsewhere (footnote 108 of ref. [33]) we have listed websites that provide information on SARS-CoV-2 wastewater-monitoring activities in at least 48 states, many of which also require clicking through multiple levels to view current data and their public health implications. Other than the direct communications that we have with DHD and our occasional in-person outreach events, we have little information about the impact of our electronic communications on others.

Moving forward, the collaborative model described here holds promise as a viable approach to screen for pathogens, analyze data, and operationalize tailored response efforts to other pathogens that threaten the public. Such programs need to balance the commonwealth benefits of WBE with potential negative impacts. The ethical framework outlined by Ram et al. [33] provides a guide for considering community benefit, health equity, privacy, and effective governance in the design, evaluation, and implementation of wastewater screening efforts. While the COVID-19 pandemic still persists, and before our next pandemic, now is the appropriate time to reinforce relationships, to discuss surveillance and response policy, and to develop ethical guidelines for future WBE efforts [63]. Guidelines regarding what type, frequency, distribution, and severity of public health threat should trigger this type of community data gathering will be important for ethical implementation or limitation of future efforts. With these resolutions and plans in place, academic, health department, and private sector partners can collaboratively meet the challenge of dynamic disease outbreaks.

## 6. Conclusions

The design of this project and its outputs should guide future collaborative relationships and practices as we consider the best ways to foster public health in the future. Collaboration among government, academic, and private sector will continue to be important in both in the terminal stages of the COVID-19 pandemic and in planning responses when other pathogens threaten our population. Many other university/health department/private sector collaborations arose early in the pandemic [27] and could be interesting to compare retrospectively to our experience in Detroit. Improvements in communication among all stakeholders, including the general public, are likely needed to build trust so that appropriate public health responses can be implemented more effectively. Further benefits include a contribution to the epidemiological understanding of this virus as well as the furnishing of public health officials with useful data to operationalize a united response [64,65].

## Data Availability

Data described in this paper are available for inspection upon request from the corresponding author, except that identification of sites for which the data source is restricted will not be shared. ddRT-PCR measurement results from all sites have been uploaded to the State of Michigan’s COVID-19 Wastewater Testing Dashboard, at https://gisportal.state.mi.us/portal/apps/insights/index.html#/view/52bbb104ed574887918f990af9f3debe (accessed, for example, on 28 June 2023), which displays data from all non-restricted sites illustrated in this paper. Other data, statistics, and derived calculations on flow and other characteristics of the wastewater samples and sites are contained in paper and/or electronic files of the project, and are available from the corresponding author upon request.

## Supplementary Materials

The following supporting information can be downloaded at: https://www.mdpi.com/article/10.3390/ijerph20176651/s1, Text, Materials and Method for SARS-CoV-2 marker detection in wastewater; Figure S1: Regression of 2 hospital sewershed levels versus 18 non-hospital sewersheds one week earlier; Figure S2: Regression of 2 hospital sewershed levels versus 18 non-hospital sewersheds two weeks earlier; Figure S3: Locations of access sites HF and HP; Table S1: Locations and populations served by non-restricted sampled sewersheds. Reference [66] is cited in the supplementary materials.

## References

[1] Jacobs J.L., Haidar G., Mellors J.W. (2023). COVID-19: Challenges of viral variants. Annu. Rev. Med..

[2] Wang S.C., Rai C.I., Chen Y.C. (2023). Challenges and recent advancements in COVID-19 vaccines. Microorganisms.

[3] Yoo S.H., Kim L., Lu M., Nagoshi K., Namchuk M.N. (2022). A review of clinical efficacy data supporting emergency use authorization for COVID-19 therapeutics and lessons for future pandemics. CTS-Clin. Transl. Sci..

[4] D'Heysselaer S.D., Parisi G., Lisson M., Bruyere O., Donneau A.F., Fontaine S., Gillet L., Bureau F., Darcis G., Thiry E. (2023). Systematic review of the key factors influencing the indoor airborne spread of SARS-CoV-2. Pathogens.

[5] Whitsel L.P., Ajenikoko F., Chase P.J., Johnson J., McSwain B., Phelps M., Radcliffe R., Faghy M.A. (2023). Public policy for healthy living: How COVID-19 has changed the landscape. Prog. Cardiovasc. Dis..

[6] Parmet W.E., Khalik F. (2023). Judicial review of public health powers since the start of the COVID-19 pandemic: Trends and implications. Am. J. Public Health.

[7] Relihan D.P., Holman E.A., Garfin D.R., Ditto P.H., Silver R.C. (2023). Politicization of a pathogen: A prospective longitudinal study of COVID-19 responses in a nationally representative US sample. Political Psychol..

[8] Zhu Y.C., Fitzpatrick M.A., Bowen S.A. (2023). Factors related to compliance with CDC COVID-19 guidelines: Media use, partisan identity, science knowledge, and risk assessment. West. J. Commun..

[9] Pan X., Chen D., Xia Y., Wu X., Li T., Ou X., Zhou L., Liu J. (2020). Viral load of SARS-CoV-2 in clinical samples. Lancet Infect. Dis..

[10] Ahmed W., Bertsch P.M., Angel N., Bibby K., Bivins A., Dierens L., Edson J., Ehret J., Gyawali P., Hamilton K. (2020). Detection of SARS-CoV-2 RNA in commercial passenger aircraft and cruise ship wastewater: A surveillance tool for assessing the presence of COVID-19 infected travelers. J. Travel Med..

[11] Galani A., Aalizadeh R., Kostakis M., Markou A., Alygizakis N., Lytras T., Adamopoulos P.G., Peccia J., Thompson D.C., Kontou A. (2022). SARS-CoV-2 wastewater surveillance data can predict hospitalizations and ICU admissions. Sci. Total Environ..

[12] Panchal D., Prakash O., Bobde P., Pal S. (2021). SARS-CoV-2: Sewage surveillance as an early warning system and challenges in developing countries. Environ. Sci. Pollut. Res..

[13] Peccia J., Zulli A., Brackney D.E., Grubaugh N.D., Kaplan E.H., Casanovas-Massana A., Ko A.I., Malik A.A., Wang D., Wang M. (2020). SARS-CoV-2 RNA concentrations in primary municipal sewage sludge as a leading indicator of COVID-19 outbreak dynamics. medRxiv.

[14] Prado T., Fumian T.M., Mannarino C.F., Resende P.C., Motta F.C., Eppinghaus A.L.F., Chagas Do Vale V.H., Braz R.M.S., De Andrade J.D.S.R., Maranhão A.G. (2021). Wastewater-based epidemiology as a useful tool to track SARS-CoV-2 and support public health policies at municipal level in Brazil. Water Res..

[15] Rossmann K., Grossmann G., Frangoulidis D., Clasen R., Munch M., Hasenknopf M., Wurzbacher C., Tiehm A., Stange C., Ho J.N. (2021). Innovative SARS-CoV-2 crisis management in the public health sector: Corona dashboard and wastewater surveillance using the example of Berchtesgadener Land, Germany. Bundesgesundheitsblatt-Gesundheitsforschung-Gesundheitsschutz.

[16] Shah S., Gwee S.X.W., Ng J.Q.X., Lau N., Koh J.Y., Pang J.X. (2022). Wastewater surveillance to infer COVID-19 transmission: A systematic review. Sci. Total Environ..

[17] Li B., Di D.Y.W., Saingam P., Jeon M.K., Yan T. (2021). Fine-scale temporal dynamics of SARS-CoV-2 RNA abundance in wastewater during a COVID-19 lockdown. Water Res..

[18] Street R., Mathee A., Mangwana N., Dias S., Sharma J.R., Ramharack P., Louw J., Reddy T., Brocker L., Surujlal-Naicker S. (2021). Spatial and temporal trends of SARS-CoV-2 RNA from wastewater treatment plants over 6 weeks in Cape Town, South Africa. Int. J. Environ. Res. Public Health.

[19] Brouwer A.F., Eisenberg J.N.S., Pomeroy C.D., Shulman L.M., Hindiyeh M., Manor Y., Grotto I., Koopman J.S., Eisenberg M.C. (2018). Epidemiology of the silent polio outbreak in Rahat, Israel, based on modeling of environmental surveillance data. Proc. Natl. Acad. Sci. USA.

[20] Wells C.R., Huppert A., Fitzpatrick M.C., Pandey A., Velan B., Singer B.H., Bauch C.T., Galvani A.P. (2020). Prosocial polio vaccination in Israel. Proc. Natl. Acad. Sci. USA.

[21] Wu F.Q., Xiao A., Zhang J.B., Moniz K., Endo N., Armas F., Bushman M., Chai P.R., Duvallet C., Erickson T.B. (2021). Wastewater surveillance of SARS-CoV-2 across 40 US states from February to June 2020. Water Res..

[22] Augusto M.R., Claro I.C.M., Siqueira A.K., Sousa G.S., Caldereiro C.R., Duran A.F.A., de Miranda T.B., Camillo L.D.B., Cabral A.D., Bueno R.D. (2022). Sampling strategies for wastewater surveillance: Evaluating the variability of SARS-COV-2 RNA concentration in composite and grab samples. J. Environ. Chem. Eng..

[23] Gerrity D., Papp K., Stoker M., Sims A., Frehner W. (2021). Early-pandemic wastewater surveillance of SARS-CoV-2 in Southern Nevada: Methodology, occurrence, and incidence/prevalence considerations. Water Res. X.

[24] Zhao L., Zou Y., Li Y., Miyani B., Spooner M., Gentry Z., Jacobi S., David R.E., Withington S., McFarlane S. (2022). Five-week warning of COVID-19 peaks prior to the Omicron surge in Detroit, Michigan using wastewater surveillance. Sci. Total Environ..

[25] Layton B.A., Kaya D., Kelly C., Williamson K.J., Alegre D., Bachhuber S.M., Banwarth P.G., Bethel J.W., Carter K., Dalziel B.D. (2022). Evaluation of a wastewater-based epidemiological approach to estimate the prevalence of SARS-CoV-2 infections and the detection of viral variants in disparate Oregon communities at city and neighborhood scales. Environ. Health Perspect..

[26] Greco M., Campagna M., Cricelli L., Grimaldi M., Strazzullo S. (2022). COVID-19-related innovations: A study on underlying motivations and inter-organizational collaboration. Ind. Mark. Manag..

[27] Harris-Lovett S., Nelson K., Beamer P., Bischel H.N., Bivins A., Bruder A., Butler C., Camenisch T.D., Long S.K.D., Karthikeyan S. (2021). Wastewater surveillance for SARS-CoV-2 on college campuses: Initial efforts, lessons learned and research needs. Int. J. Environ. Res. Public Health.

[28] West N.W., Vasquez A.A., Bahmani A., Khan M.F., Hartrick J., Turner C.L., Shuster W., Ram J.L. (2022). Sensitive detection of SARS-CoV-2 molecular markers in urban community sewersheds using automated viral RNA purification and digital droplet PCR. Sci. Total Environ..

[29] Ram J.L., Thompson B., Turner C., Nechvatal J.M., Sheehan H., Bobrin J. (2007). Identification of pets and raccoons as sources of bacterial contamination of urban storm sewers using a sequence-based bacterial source tracking method. Water Res..

[30] Hillary L.S., Farkas K., Maher K.H., Lucaci A., Thorpe J., Distaso M.A., Gaze W.H., Paterson S., Burke T., Connor T.R. (2021). Monitoring SARS-CoV-2 in municipal wastewater to evaluate the success of lockdown measures for controlling COVID-19 in the UK. Water Res..

[31] Amereh F., Negahban-Azar M., Isazadeh S., Dabiri H., Masihi N., Jahangiri-Rad M., Rafiee M. (2021). Sewage systems surveillance for SARS-CoV-2: Identification of knowledge gaps, emerging threats, and future research needs. Pathogens.

[32] Gable L., Ram N., Ram J.L. (2020). Legal and Ethical Implications of Wastewater SARS-CoV-2 Monitoring for COVID-19 Surveillance, Supplement. J. Law Biosci..

[33] Ram N., Gable L., Ram J.L. (2022). The future of wastewater monitoring for the public health. Univ. Richmond Law Rev..

[34] Water Research Foundation (2020). Understanding the Factors That Affect the Detection and Variability of SARS-CoV-2 in Wastewater. https://www.waterrf.org/sites/default/files/file/2020-07/RFQ_5093.pdf.

[35] Quinn K.L., Abdel-Qadir H., Barrett K., Bartsch E., Beaman A., Biering-Sørensen T., Colacci M., Cressman A., Detsky A., Gosset A. (2022). Variation in the risk of death due to COVID-19: An international multicenter cohort study of hospitalized adults. J. Hosp. Med..

[36] Lau-Ng R., Caruso L.B., Perls T.T. (2020). COVID-19 deaths in long-term care facilities: A critical piece of the pandemic puzzle. J. Am. Geriatr. Soc..

[37] Shen K. (2022). Relationship between nursing home COVID-19 outbreaks and staff neighborhood characteristics. PLoS ONE.

[38] Gable L., Ram N., Ram J.L. (2020). Legal and ethical implications of wastewater SARS-CoV-2 monitoring for COVID-19 surveillance. J. Law Biosci..

[39] Michigan.gov (2022). Coronavirus/Michigan Data. Long Term Care Data. https://www.michigan.gov/coronavirus/0,9753,7-406-98163_98173-526911--,00.html.

[40] Zhou Y., Zhi H., Teng Y. (2022). The outbreak of SARS-CoV-2 Omicron lineages, immune escape, and vaccine effectivity. J. Med. Virol..

[41] Oloye F.F., Xie Y.W., Asadi M., Cantin J., Challis J.K., Brinkmann M., McPhedran K.N., Kristian K., Keller M., Sadowski M. (2022). Rapid transition between SARS-CoV-2 variants of concern Delta and Omicron detected by monitoring municipal wastewater from three Canadian cities. Sci. Total Environ..

[42] Wilhelm A., Agrawal S., Schoth J., Meinert-Berning C., Bastian D., Orschler L., Ciesek S., Teichgräber B., Wintgens T., Lackner S. (2022). Early detection of SARS-CoV-2 Omicron BA.4 and BA.5 in German wastewater. Viruses.

[43] Betancourt W.Q., Schmitz B.W., Innes G.K., Prasek S.M., Pogreba Brown K.M., Stark E.R., Foster A.R., Sprissler R.S., Harris D.T., Sherchan S.P. (2021). COVID-19 containment on a college campus via wastewater-based epidemiology, targeted clinical testing and an intervention. Sci. Total Environ..

[44] Betancourt W.W., Schmitz B.W., Innes G.K., Pogreba Brown K.M., Prasek S.M., Stark E.R., Foster A.R., Sprissler R.S., Harris D.T., Sherchan S.P. (2020). Wastewater-based epidemiology for averting COVID-19 outbreaks on the University of Arizona campus. medRxiv.

[45] Centers for Disease Control and Prevention (CDC) US Dept. of Public Health Services (2020). 2019-Novel Coronavirus (2019-nCoV) Real-Time rRT-PCR Panel Primers and Probes, Division of Viral Diseases, National Center for Immunization and Respiratory Diseases. https://www.cdc.gov/coronavirus/2019-ncov/downloads/rt-pcr-panel-primer-probes.pdf.

[46] Medema G., Heijnen L., Elsinga G., Italiaander R., Brouwer A. (2020). Presence of SARS-Coronavirus-2 RNA in sewage and correlation with reported COVID-19 prevalence in the early stage of the epidemic in the Netherlands. Environ. Sci. Technol. Lett..

[47] Wu F., Zhang J., Xiao A., Gu X., Lee W.L., Armas F., Kauffman K., Hanage W., Matus M., Ghaeli N. (2020). SARS-CoV-2 Titers in Wastewater Are Higher than Expected from Clinically Confirmed Cases. mSystems.

[48] Falzone L., Musso N., Gattuso G., Bongiorno D., Palermo C.I., Scalia G., Libra M., Stefani S. (2020). Sensitivity assessment of droplet digital PCR for SARS-CoV-2 detection. Int. J. Mol. Med..

[49] Flood M.T., D’Souza N., Rose J.B., Aw T.G. (2021). Methods evaluation for rapid concentration and quantification of SARS-CoV-2 in raw wastewater using droplet digital and quantitative RT-PCR. Food Environ. Virol..

[50] Smith K. (2022). Is Michigan Prepared for the Next COVID-19 Surge? Wastewater Testing May Help. WXYZ.

[51] McClary-Gutierrez J.S., Mattioli M.C., Marcenac P., Silverman A.I., Boehm A.B., Bibby K., Balliet M., de los Reyes F.L., Gerrity D., Griffith J.F. (2021). SARS-CoV-2 Wastewater Surveillance for Public Health Action. Emerg. Infect. Dis..

[52] Geurts A., Geerdink T., Sprenkeling M. (2022). Accelerated innovation in crises: The role of collaboration in the development of alternative ventilators during the COVID-19 pandemic. Technol. Soc..

[53] Hrudey S.E., Bischel H.N., Charrois J., Chik A.H.S., Conant B., Delatolla R., Dorner S., Graber T.E., Hubert C., Isaac-Renton J. (2022). Wastewater surveillance for SARS-CoV-2 RNA in Canada. Facets.

[54] Sims N., Kasprzyk-Hordern B. (2020). Future perspectives of wastewater-based epidemiology: Monitoring infectious disease spread and resistance to the community level. Environ. Int..

[55] Centers for Disease Control and Prevention (2023). COVID Data Tracker. https://covid.cdc.gov/covid-data-tracker/#wastewater-surveillance.

[56] Wartell B.A., Proano C., Bakalian L., Kaya D., Croft K., McCreary M., Lichtenstein N., Miske V., Arcellana P., Boyer J. (2022). Implementing wastewater surveillance for SARS-CoV-2 on a university campus: Lessons learned. Water Environ. Res..

[57] Bhatnagar S., Kumar S., Rathore P., Sarma R., Malhotra R.K., Choudhary N., Thankachan A., Haokip N., Singh S., Pandit A. (2021). Surviving COVID-19 is half the battle; living life with perceived stigma is other half: A cross-sectional study. Indian J. Psychol. Med..

[58] Smith-Morris C. (2017). Epidemiological placism in public health emergencies: Ebola in two Dallas neighborhoods. Soc. Sci. Med..

[59] Price M., Trowsdale S. (2022). The ethics of wastewater surveillance for public health. J. Hydrol. N. Z..

[60] Spurbeck R.R., Minard-Smith A., Catlin L. (2021). Feasibility of neighborhood and building scale wastewater-based genomic epidemiology for pathogen surveillance. Sci. Total Environ..

[61] Shrestha S., Yoshinaga E., Chapagain S.K., Mohan G., Gasparatos A., Fukushi K. (2021). Wastewater-based epidemiology for cost-effective mass surveillance of COVID-19 in low- and middle-income countries: Challenges and opportunities. Water.

[62] Naughton C.C., Holm R., Lin N.J.J., James B.P., Smith T. (2023). Online dashboards for SARS-CoV-2 wastewater data need standard best practices: An environmental health communication agenda. J. Water Health.

[63] Ram N., Shuster W., Gable L., Ram J.L. (2023). Ethical and legal wastewater surveillance. Science.

[64] Khoury M.J., Armstrong G.L., Bunnell R.E., Cyril J., Iademarco M.F. (2020). The intersection of genomics and big data with public health: Opportunities for precision public health. PLoS Med.

[65] Khoury M.J., Bowen S., Dotson W.D., Drzymalla E., Green R.F., Goldstein R., Kolor K., Liburd L.C., Sperling L.S., Bunnell R. (2022). Health equity in the implementation of genomics and precision medicine: A public health imperative. Genet. Med..

[66] Vasquez A.A., West N.W., Bahmani A., Ram J.L. (2021). Rapid and Direct Method to Extract SARS-CoV-2 RNA from Municipal Wastewater Using the CHEMAGIC 360™ 12-Rod Head Platform.

