# Supplementary material for: Wastewater Monitoring for Infectious Disease: Intentional Relationships between Academia, the Private Sector, and Local Health Departments for Public Health Preparedness
Source: Int J Environ Res Public Health. 2023 Aug 25;20(17):6651. doi: 10.3390/ijerph20176651 (PMC10487196; doi:10.3390/ijerph20176651)
Supplement: Supplementary file 1 [file ijerph-20-06651-s001.zip › ijerph-2320991-supplementary.pdf]

Supplement to:

Wastewater monitoring for infectious disease: Intentional relationships between academia, the private sector, and local health departments for public health preparedness

Jeffrey L. Ram, William Shuster, Lance Gable, Carrie L. Turner, James Hartrick, Adrian A. Vasquez, Nicholas W, West, Azadeh Bahmani, and Randy E. David

1. Materials and Methods for SARS-CoV-2 marker detection in wastewater

Wastewater samples were collected from small (estimated populations of 150 to 3,000 people) to medium-sized (3,000 to 50,000 people) sewersheds in the City of Detroit. Some of the sewersheds were the drainage of a single building (e.g., Wayne State University dormitories) while other small sewersheds consisted of a focal institution (e.g. a long-term care facility or a detention facility, which may have 100 – 500 residents and staff) and its surrounding neighborhood ranging in size up to 3,000 people. In 2021, with funding and collaboration from the Michigan Department of Health and Human Services (MDHHS) and the DHD, the WSU team, and LimnoTech were able to lead the expansion of the project to the entire city of Detroit by developing a municipality-wide sampling plan designed to accumulate the most relevant and actionable public health data. In this project, supported by MDHHS (2021-2023), WSU provided timely measurements of SARS-CoV-2 in wastewater and other parameters from 20 sewersheds across the city of Detroit (Figure 1). In brief, the field protocol involved weekly sampling on a Monday or Tuesday, conducted at the same time and on the same day each week, except during weeks with holidays when sampling may be delayed one day. Samplings were performed in the morning, catching the morning diurnal peak of wastewater production between 8 - 10 AM. Wastewater was collected as a grab sample at each, a field blank was collected approximately every tenth sites randomly distributed, and then water quality indicators (pH, temperature, dissolved oxygen, conductivity, turbidity) were measured, along with flow at a subset of the sites. If viral RNA copies per unit volume were high at a given site or set of sites, a second follow-up sample within the same week might be collected and analyzed from the same sites. Sewershed wastewater flows were accessed via manholes and collected as grab samples in clean, unused plastic bottles of either 250 mL or 500 mL size placed via extension pole into the sewage stream at depths of 2 m to 13 m below street level. “Field blank” water samples were collected approximately once every 10 samples from pure water brought to the field and transferred to a collection bottle in the field at a randomly selected collection site. Samples were stored in a cooler on ice until delivered with a chain of custody form to the analysis laboratory at Wayne State University School of Medicine within two hours of collection.

Extraction and purification of nucleic acids from wastewater samples utilized an automated magnetic bead procedure based on equipment and reagents for the Perkin-Elmer

ChemagicTM-360. Processing usually began within one hour of delivery of the sample to the laboratory but samples may have been stored at 4°C for up to 4 days prior to processing. The processing procedure has been described in detail in a published protocol (Vasquez et al. 2021) and analysis study (West et al. 2022). Briefly, 45 mL of a well-mixed sample was centrifuged at 5,000 x g at 4 °C for 15 min; 10 mL of supernatant was transferred and incubated at 55 °C with carrier RNA (Poly A RNA, 7 μL PerkinElmer CMG842), Proteinase K (50 μL PerkinElmer CMG749), and lysis buffer 1 (8 mL PerkinElmer CMG749), and an added extraction control (Phi6 phage, 10 μL of 10^6 pfu/mL) for 30 min; magnetic beads (50 μL PerkinElmer CMG749) were then added and the prepared sample was then processed in the Chemagic™-360 as controlled by the Chemagic™Viral10k 360 H12 prefilling drying VD210119.che protocol, yielding purified nucleic acids in 85 μL elution buffer within 75 min. Typical runs included 11 sewage samples and the field blank collected the same day processed simultaneously, including the Phi6 extraction control, with the sewage samples.

The amount of SARS-CoV-2 markers and Phi6 in the purified eluate was determined for each sample in triplicate by digital droplet PCR on a Bio-Rad platform (Bio-Rad QX200 Automated Droplet Generator, Bio-Rad C1000 ThermoCycler, and QX200 Droplet Reader). Reactions were set up in 20 μL (including 5 μL of the Chemagic-purified template eluate) from which 10,000 – 20,000 nL sized droplets were generated and amplified. Primers and probes targeted the N1 and N2 sequences of SARS-CoV-2 (duplex reaction with FAM and HEX labeled probes) and the Phi6 spiked-in internal standard (FAM-labeled probe), as previously described (Table 1 of West et al. (2022)). ddPCR controls included a negative control (elution buffer used in place of template), a ddPCR Phi6 quantitative control (10 μL of 10^6 pfu/mL added to 75 μL elution buffer), and a SARS-CoV-2 positive control, in addition to the field blank control, as previously described.

Interpretation of positive and negative droplets as target copies/reaction is based on Poisson statistics, utilizing the Bio-Rad QuantaSoft software package version 1.7.4.0917, and the number of copies per reaction was then used to calculate the number of copies/100 mL in the original wastewater solution. The limit of detection corresponds to 2 positive droplets (i.e., data are not reported quantitatively unless based on 3 or more positive droplets), as recommended by Bio-Rad. For the wastewater volumes, purification procedures, and reaction volumes described here, this corresponds to a limit of detection of 600 copies of the N1 and N2 markers/100 mL of wastewater. Droplet fluorescence of positive droplets indicates that ddPCR reactions are not inhibited from reaching digital droplet fluorescence endpoints for any site. As reported in previous studies using this procedure (West et al., 2022), recovery of RNA viruses from wastewater samples with this procedure varies systematically between different sites; those studies also showed that Phi6 spiked directly into the ddPCR purified sewage reaction mix reveals no ddPCR inhibition in comparison to the Phi6-eluate spiked control.

Resulting data, expressed in copies of N1 and N2/100 mL were then organized using Excel pivot tables, statistical programs within Excel or, as needed, from GraphPad Prism (GraphPad Software LLC, Version 9.4.1), and graphics software within both of those programs.

2. Geographic and estimated population sizes for non-restricted sewersheds

Due to the small size of the sewersheds associated with long-term care facilties and other congregate living sites, we agreed not to identify the precise location of their associated sampling sites. Other sites for which we have not placed public restrictions, corresponding to the sites with two letter labels in Figure 1, are the following, provided with exact locations of the sampling manhole (latitude and longitude) and estimated populations served.

Table S1. Locations and popultions served by non-restricted sampled sewersheds

Sewershed ID	site type[1]	zipcode of sewershed	manhole Latitude & Longitude*	Estimated Population Served	Comments
AS	WSU-D	48202	42.355477,-83.072155	420	dormitory
CT*/HT	ZIP	48214	42.35003, -82.99918	3,896	HT is easier to collect, health care & residential
DB	HOSP	48201	42.34761, -83.05102	2,000	staff & patients, <150 residents
HF*/HP	ZIP	48235	42.410251, -83.189422	23,500	HP probably collected only part of the population
HH	HOSP	48201	42.35089, -83.05898	1,000	staff & patients
PA	ZIP	48228	42.3361-83.2169	4,517	residential
SG	ZIP	48205	42.43516, -82.97638	12,289	businesses and residential
UC	WSU-D	48201	42.352389-83.062331	295	dormitory; also includes daycare & radio station
WG	WSU-D	48202	42.355922, -83.072581	415	dormitory
WH	WSU-D	48202	42.357428, -83.073664	150	dormitory and campus health center

[1]site types: WSU-D, Wayne State dormitory; ZIP, ZIP Code-associated; HOSP, hospital

*The collection site was changed (see text) . The latitude and longitude are given for the ID name with the *.

3. Linear regression of SARS-CoV-2 detected in 2 hospital sewersheds versus 18 sewersheds city-wide in Detroit, MI

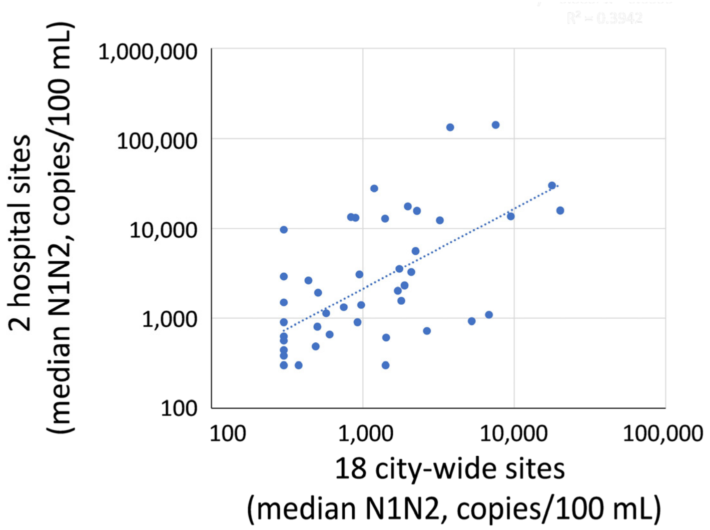

Figure S1. Regression of the log10 (median) of 2 hospital sewersheds levels versus the log10 (median) levels of 18 non-hospital sewersheds in Detroit one week earlier. R^2=0.39, p<0.001, linear regression by Excel data analysis plug-in.

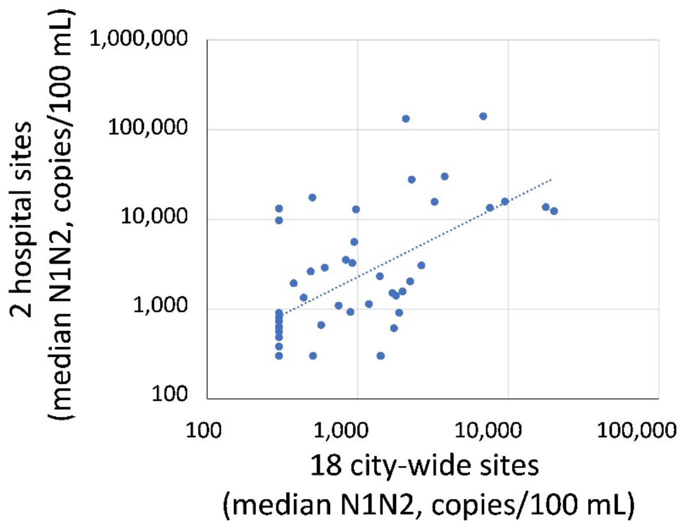

Figure S2. Regression of the log10 (median) of 2 hospital sewersheds levels versus the log10(median) levels of 18 non-hospital sewersheds in Detroit two weeks earlier. R^2=0.35, p<0.001, linear regression by Excel data analysis plug-in.

4. Map showing locations of access sites HF and HP, for sampling from ZIP Code 48235

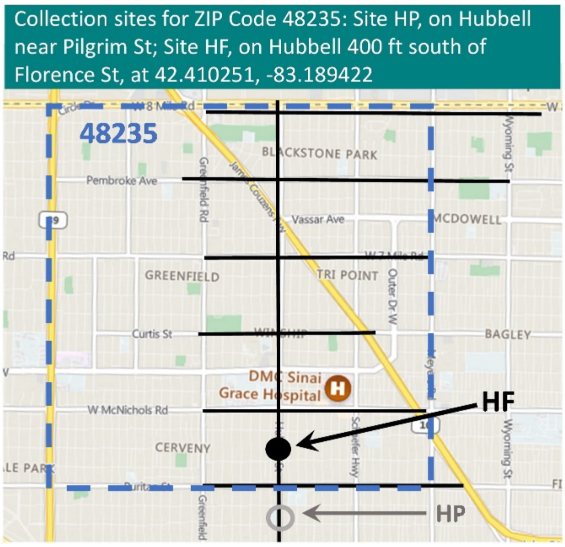

Figure S3. Map showing the change in sampling site for ZIP Code 48235 from site HP to HF. Data in Figure 6 during the Delta and Omicron outbreaks show that SARS-CoV-2 measurements from HP were unexpectedly low and that collections from HF correlated better

than HP with two other ZIP Code-associated sewersheds, SG and PA. The dashed blue box shows the boundaries of ZIP Code 48235; black lines indicate the sewers.

Vasquez AA, West NW, Bahmani A, Ram JL. 2021. Rapid and direct method to extract SARS-CoV-2 RNA from municipal wastewater using the Chemagic 360™ 12-rod head platform. https://dx.doi.org/10.17504/protocols.io.b2reqd3e.

West NW, Vasquez AA, Bahmani A, Khan MF, Hartrick J, Turner CL, Shuster W, Ram JL. 2022. Sensitive detection of SARS-CoV-2 molecular markers in urban community sewersheds using automated viral RNA purification and digital droplet PCR. The Science of the total environment 847: 157547-157547.
